# The hospital at home in the USA: current status and future prospects

**DOI:** 10.1038/s41746-024-01040-9

**Published:** 2024-02-27

**Authors:** Jay A. Pandit, Jeff B. Pawelek, Bruce Leff, Eric J. Topol

**Affiliations:** 1 Scripps Translational Research Institute, Scripps Research, La Jolla, CA USA; 2 Department of Medicine, Johns Hopkins University School of Medicine, Baltimore, MD USA

**Keywords:** Health services, Diagnostic markers, Health care economics, Health policy

## Abstract

The annual cost of hospital care services in the US has risen to over $1 trillion despite relatively worse health outcomes compared to similar nations. These trends accentuate a growing need for innovative care delivery models that reduce costs and improve outcomes. HaH—a program that provides patients acute-level hospital care at home—has made significant progress over the past two decades. Technological advancements in remote patient monitoring, wearable sensors, health information technology infrastructure, and multimodal health data processing have contributed to its rise across hospitals. More recently, the COVID-19 pandemic brought HaH into the mainstream, especially in the US, with reimbursement waivers that made the model financially acceptable for hospitals and payors. However, HaH continues to face serious challenges to gain widespread adoption. In this review, we evaluate the peer-reviewed evidence and discuss the promises, challenges, and what it would take to tap into the future potential of HaH.

## Introduction

Prior to the 19th century, hospitals in the United States (US) primarily served poor and marginalized communities while the upper class received medical and surgical care at home^1^. The mid to late 19th century brought about the technological sophistication of medical practice and the professionalization of healthcare, notably nursing, which reshaped “the day-to-day texture of hospital life” and improved clinical outcomes, thereby driving the transition from acute home care to hospital care^2^. The formation of academic medical centers and centralization of emerging medical technologies cemented the hospital’s role as the primary site of care for sick patients by the 20^th^ century^3^^,^^4^. However, record-high hospital expenditures and increasing patient volumes has not necessarily translated into better health outcomes, spotlighting problems associated with inpatient hospital stays, such as nosocomial infection, iatrogenic complications, and medical errors^5–10^. With over $1 trillion spent on hospital services in 2021 and projected increases in the coming years, there is an urgent need to explore more efficient care delivery models^11^. Growing evidence and advancements in digital health technologies have positioned the Hospital at Home (HaH) model as a promising strategy to reduce health care spending and improve patient outcomes. A recent comprehensive meta-analysis of randomized trials of HaH versus hospital care demonstrated lower risk of readmission or long-term care admission and higher patient satisfaction with at-home acute care^12^. This begs the question: does the hospital need to be the primary venue of care for those who aren’t critically ill?

Out of necessity rather than intent, the COVID-19 pandemic tested this hypothesis. The pandemic presented hospitals with the challenge of caring for (and getting reimbursed for) sick patients while limiting admissions to only those who required critical care. While prior attempts to utilize home-based care to shorten hospital stays and reduce hospital admissions had already demonstrated clinical utility, in the US there was a scarcity of adoption due to lack of perceived need, compelling evidence of safety, challenges in altering health care delivery, or supportive reimbursement models^13–15^. The absence of sustained strategies to reduce hospital admissions after the peak of the COVID-19 pandemic reveals some of the obstacles that must be addressed for HaH models to gain wider acceptance in the US. Globally, HaH studies have reported noninferior and even superior patient outcomes, higher quality of life, and cost effectiveness compared to inpatient care for select clinical conditions^16–23^. Notably, in countries like Australia and Norway, in-person at-home acute care has gained significant traction and has been offered in most hospitals for over 15 years^24,25^.

Regardless of adoption, the primary application for at-home care programs continues to be management of chronic disease and the primary intervention remains in-person clinical staff with a gradual implementation of virtual care, and despite the availability, there has not been much evaluation of digital remote monitoring tools even though there was rampant use for acute care delivery during the COVID-19 pandemic. Further, a new crop of health technology and care delivery companies has risen to help health systems set up HaH programs, utilizing care delivery approaches ranging from in-person to completely virtual. In this manuscript we provide an assessment of the major US published and ongoing studies in the field of HaH, highlighting the promises and challenges that HaH programs face to scale and become a mainstream alternative form of acute care delivery. For the purposes of this manuscript, we defined HaH as fully substituting acute inpatient hospital admission with at-home care, whether it be delivered by in-person, virtual or hybrid (i.e., in-person with virtual elements) care models.

## Historical context

The HaH model is not a new one. The first published trials come from the United Kingdom in the late 1970s. The first US-based prospective study of providing hospital-level care in the home, in which physicians made in-person visits, was published in the late 1990s^26^ (Supplementary Table 1). This single-site study demonstrated the feasibility, safety, and cost-effectiveness of an HaH program in participants >65 in four different acute medical conditions requiring hospital care (community acquired pneumonia (CAP), exacerbations of chronic heart failure (CHF) and chronic obstructive pulmonary disease (COPD) and cellulitis).

### In-person

This early foray in US HaH was characterized by a reliance on a clinician’s physical presence in the home. In the initial HaH study^26^, after a patient was deemed appropriate for admission by an emergency room physician, the patient was consented, sent home in an ambulance and unless the patient refused, a nurse was present to provide 1:1 care for the first 24 h, then every 8 h, followed by as needed. A HaH physician did at least daily in-person visits and determined the frequency of monitoring as well as appropriateness for discharge. These visits were found to be 40% cheaper than an inpatient hospitalization and with higher patient satisfaction^26^. To ensure the results were not provider/site specific, a follow-up national study included 4 facilities (3 Medicare managed care entities and 1 by the Veterans Health Administration)^27^. In this study, patients were given a choice between an inpatient hospital admission or acute care at home in a HaH model after an emergency physician had determined they needed to be admitted; most participants selected HaH. The HaH group had a shorter duration of acute care, fewer procedures, less in-home medical devices, fewer complications, and higher levels of satisfaction at a lower cost with better clinical outcomes^27^. Subsequently, between 2005 and 2009, several separate publications reported on patient satisfaction, functional outcomes, caregiver stress, and HaH program costs^28–31^. All reports showed favorable results for the HaH cohorts compared to hospitalized controls, acknowledging limitations due to selection bias, absence of randomization, and data missingness.

### Telemedicine

With increasing telemedicine acceptance there was a move from in-person to telemedicine via phone or video visit. In 2004, during the nascent stages of globalizing high-speed internet, a small prospective study on HaH solely using telemedicine showed that out of 25 study participants who required hospital admission (but not intensive care unit admission) for community acquired pneumonia, cellulitis, or urinary tract infection, 8 avoided index hospital admission and received 100% of their acute care at home. The other 17 were admitted to the hospital with early transfer to HaH to complete their hospitalization at home^32^, demonstrating the feasibility of HaH by telemedicine. The duration of acute care, readmissions, and costs were all lower in the HaH group compared to matched controls of hospitalized patients^32^.

In a typical study^32^, the patient’s set up required a nearby telephone outlet, and a second member of the telemedicine team communicated with the patient from a central station at the hospital. The patient was taught to obtain their vital signs using a blood pressure cuff, a stethoscope on the chest, and a pulse oximeter on the finger. Note that the technology required to do this had already been around for decades, the only novelty was virtual communication of biometrics performed either by a care provider or the patient themselves. Interestingly, despite the introduction of the smartphone, medical apps, electronic health records (EHR), and wearable sensors for biometric monitoring, it was not until 2015 that their potential was evaluated for HaH care^33^.

## From analog to digital

The remote patient monitoring movement, which began in the 1970s (STARPAHC program), had already seen an evolution of novel sensors that could collect biometrics longitudinally with high fidelity^34^. Initially it was activity trackers with step count and heart metrics in the early 2000s, transmitting data via Cellular to the cloud or Bluetooth to their respective apps^35–37^. Since then, the adoption of wearables has continued to grow exponentially^38^ covering everything from internal physiologic biometrics to external exposome measures. Given the need for higher frequency monitoring in acute care with optimal patient adherence to ensure high-level data quality, HaH programs provided a natural entry point for device and software manufacturers. The first HaH study to utilize wireless biometrics came in 2015 and performed a virtual physician visit in 50 HaH patients compared it to 52 hospitalized controls. To emphasize the significance of scalability, the study suggested that a scalable substitutive model of HaH using biometrically enhanced 2-way tele-video, virtual physician visits were safe, feasible, and highly satisfactory^33^. The primary change was 2-way communication, the use of wireless devices to collect vital signs, and a circumscribed period of 34 days (including the hospital care admission at home and then followed by 30 days of post-acute care at home). The vital signs were collected from the devices and then uploaded to a third-party website, circumventing the need for a care provider to collect and manually upload the numbers into the care portal to be reviewed. This approach shifted the burden of data entry away from the provider and the patient to the device.

### Remote Patient Monitoring (RPM) sensors

With the plethora of FDA cleared or CE marked sensors collecting different biomarkers, remote patient monitoring companies had the option to include multiple devices or find devices with multiple capabilities or work with companies that aggregated different biometric signals. Largely, wearables can be divided into biophysical or biochemical sensors^39^. Biophysical sensors use photoplethysmography, acoustic, mechanical, electrical, bioimpedance, thermal, and other signals to derive biometrics like heart rate, blood pressure, respiratory rate, temperature, pulse oximetry, activity, gait, sleep, heart rhythm, hydration, brain activity, muscle activity, and other metrics. Biochemical sensors on the other hand utilize a combination of noninvasive or minimally invasively derived biofluids to measure biofluid chemistry such as glucose, electrolytes, vitamins, lactic acid, creatinine, alcohol, urea, levodopa, cortisol, and other biomarkers. With time, sensor forms have evolved from the commonly accepted wrist bands, weighing scales, and finger clips to chest or arm patches, contact lenses, mouth guards, necklaces, and even tattoos^40–42^. Prior to the COVID-19 pandemic, the medical wearable sensor ecosystem had grown substantially, however, other than some ambulatory electrocardiography and glucose measurement devices, most of these novel sensors were left out of diagnostic tools and acute care management and were mostly utilized by Quantified Self enthusiasts with some momentum towards chronic care management^43^.

### Remote patient monitoring platforms

With the increasing number of sensors generating data, there was a need for remote patient monitoring companies to aggregate and harmonize this data for return of information to the user and other stakeholders. Device manufactures with single or multiparameter capabilities built out their own care platforms. Additionally, large consumer wrist wearable companies like Apple and Google released their own research kits^44,45^. Patients were able to connect different personal health monitoring apps that separately collected different data streams that fed into the research kits, enabling applications in wellness and athletic performance monitoring as well as offering a glimpse into chronic disease management^46,47^. However, despite the potential, there was not much implementation in the HaH space, largely due to missing design features specific to HaH use cases, inadequate backend algorithms to address signal noise, and limited EHR integration.

## Virtual care

Payors have been slowly adapting to the idea of virtual care delivery, but only for primary care and chronic conditions. From the provider side, EHR systems were allowing third party developers and EHR integration companies to facilitate health system integration, which in most cases used scanned lists of biometrics^48^. Companies like Amwell and Teladoc laid inroads facilitating hybrid (in-person and virtual) care models, but the applications continued to remain limited, such as specialist consultations, particularly in specialties which required visual pattern recognition (such as dermatology and radiology) and mostly in rural communities.

**Fig. 1:**
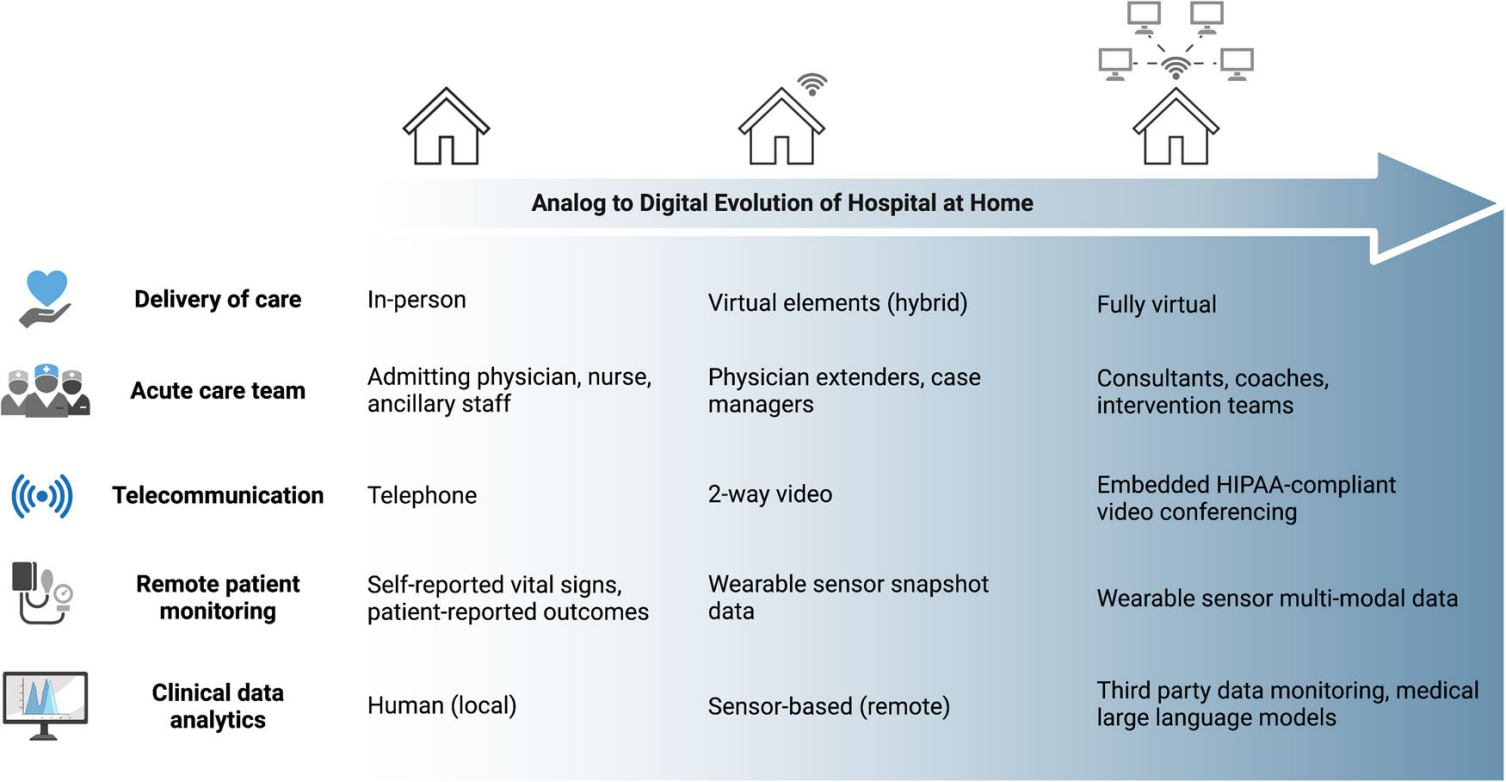
Analog to digital evolution of hospital at home. From left to right, the expansion of assets and advancements in technologies continue to evolve for acute care hospital at home. Physician extenders include providers such as nurse practitioners and physician assistants. Intervention team includes several types of medical professionals such as paramedics, physical therapists, and phlebotomists.

The idea of providing care through a 2-way virtual platform was already catching on prior to COVID-19, especially for primary care with the growth of companies like Teladoc (founded in 2002), Carbon Health (founded in 2015), Firefly health (founded in 2016), and others. Additionally, condition-specific virtual platforms like Livongo (founded in 2008) and Omada (founded in 2011) were on the rise, and the idea of having a virtual provider on-demand for a wellness or low acuity visit was catching on. However, prior to COVID-19, only a few companies were attempting to support virtual at-home acute care like Current Health (founded in 2014), Medically Home (founded in 2016), DispatchHealth (founded in 2013) with an at-home urgent care as well as Biofourmis (founded in 2015) and Heartbeat Health (founded in 2016) which were aimed at providing virtual specialty care. The modern HaH model has several core elements, all of which have significantly evolved over the last 20 years (Fig. 1).

### COVID-19 and acute care at home

With the global pandemic requiring mandatory shutdowns and the overwhelming shortage of hospital beds, virtual medical care (at the time primarily pigeon-holed into rural or remote care) rapidly became mainstream. This situation was made significantly more palatable for hospitals once most payors agreed to reimburse virtual visits^13^. Reimbursement was further expanded when an Emergency Use Authorization (EUA) waiver of certain regulations began on November 25, 2020, supporting the AHCaH initiative. Prior to this waiver, the Centers for Medicare, and Medicaid Services required around-the-clock presence of a registered nurse for parity in reimbursement for a patient admitted to an HaH program. This requirement was waived by the EUA waiver, and as of July 2023, 125 health systems and 290 hospitals in 37 states have been approved for the waiver.

Decentralized clinical studies had already demonstrated the use of wearables to identify COVID-19 infection^49,50^. Therefore, device and vital sign monitoring companies pivoted to the diagnosis and monitoring of COVID-19 patients^51^. Hospitals relied on video calling platforms like Zoom, Microsoft Teams, Skype, Twilio and Whatsapp, eventually leading to partnerships like the ones between Microsoft and Amwell or Doximity and Twilio^52^. As volumes increased, communications companies turned into large telehealth companies. Ultimately, the remote/virtual first option gained popularity and companies started looking to incorporate novel sensors like home physical exam devices in different forms in lieu of the in-person physical exam, leading to large-scale mergers like Livongo and Teladoc, Best Buy and Current Health, and Amazon and One Medical^53^.

The clinical need to manage COVID positive patients at home was evident. Studies demonstrated the potential of HaH programs to increase hospital surge capacity, reduce nosocomial spread of the virus, and ultimately provide noninferior acute care at home in acutely ill COVID patients^54–56^. The need to provide continuous monitoring with a user-friendly platform created a doorway for technology companies to enter the HaH space with remote wearable technologies. The Medically Home and Kaiser Permanente partnership began before COVID -19 to treat a range of conditions, and thus was well-positioned to treat over 2000 COVID positive patients at home. The program subsequently expanded to cover other conditions like acute exacerbations of COPD exacerbation, CHF and infections like pneumonia, urinary tract infection and cellulitis^57^. HaH companies have raised millions of dollars demonstrating their feasibility and validating their care delivery approach, yet the field is nascent, and we hope that large-scale prospective evaluation of clinical outcomes beyond COVID-19 are soon to come.

## Challenges

The Centers for Medicare and Medicaid Services AHCaH waiver that catalyzed the creation of many HaH programs is set to expire in late 2024^58^, and while we have highlighted the many promises of HaH programs, here we present the consistently cited challenges that need to be addressed before HaH programs can cement their role in care delivery.

### Evidence

To date we have identified 7 HaH studies utilizing digital sensors, platforms, and mostly virtual care evaluating the clinical outcomes of acute care HaH programs^56,59–64^. Three of these studies demonstrated the ability to provide COVID-19 acute hospital care in the home and increase surge capacity without adverse outcomes. A retrospective study on 1031 participants admitted for acute respiratory disease compared HaH care versus hospital care and found that HaH was safe and effective for patients in the 30day period post discharge^61^. A randomized controlled trial of 172 HaH patients showed virtual physician visits were non-inferior to in-home physician visits^62^. One study that looked at hybrid care (in person plus virtual care) of 679 acutely ill patients in Florida and Wisconsin with the use of a single command center demonstrated that the Acute Care at Home Hybrid model was able to provide high acuity care and post-acute care without major difference in length of stay, mortality, discharge, and readmission metrics^64^.

Payors and regulatory authorities continue to ask for more compelling evidence, with more studies looking at comparisons between different forms of care delivery (in-person to completely virtual) looking at endpoints of morbidity, mortality as well as cost effectiveness and quality of care measures. Additionally, there is a need to define the ideal HaH model including equipment requirements. We need to extend beyond the 30-day period, which is one of Centers for Medicare and Medicaid Services criteria for readmission penalties, and look at the 90 day period^65^. As the field matures, the capability of technology will eventually advance predictive analytics to intervention and therapeutics, and these will need to be evaluated as well. As large hospital systems continue to build HaH programs, two things are clear: the HaH movement has demonstrated feasibility, efficacy, and effectiveness; and, if given the choice, many patients would select it. However, it remains to be seen how best to customize HaH treatment, and whether remote patient monitoring needs a more individualized approach based on each patient’s clinical presentation, social support, and other demographic factors.

### Analytical

In the era of digital sensors there is an ocean of possible multimodal snapshot, episodic, and continuous data available to inform clinical insight^66^. Ingesting this data, harmonizing it, building pipelines to share and analyze it, and turning it into clinically actionable insight presents a major data quality challenge. A master clinician typically does this in the hospital, using vital signs, imaging, prior history, and biomarkers to determine a management plan. Today, we have computing and processing power to automate this as evidenced by large language models passing medical licensing exams and the concept of general medical artificial intelligence (GMAI)^67^. The HaH program provides a potential test model given its relatively short time frame, continuity with a hospitalization, and multiple touchpoints. Artificial intelligence is currently focused on making repetitive tasks more efficient, but with efforts to collect, adjudicate and harmonize multimodal data and inclusive pre-training, it is reasonable to expect the future development of an AI-based tool to support remote medical decision making, adverse event prediction and detection, and patient education to help develop trust in novel models of care delivery.

### Structural

**Fig. 2:**
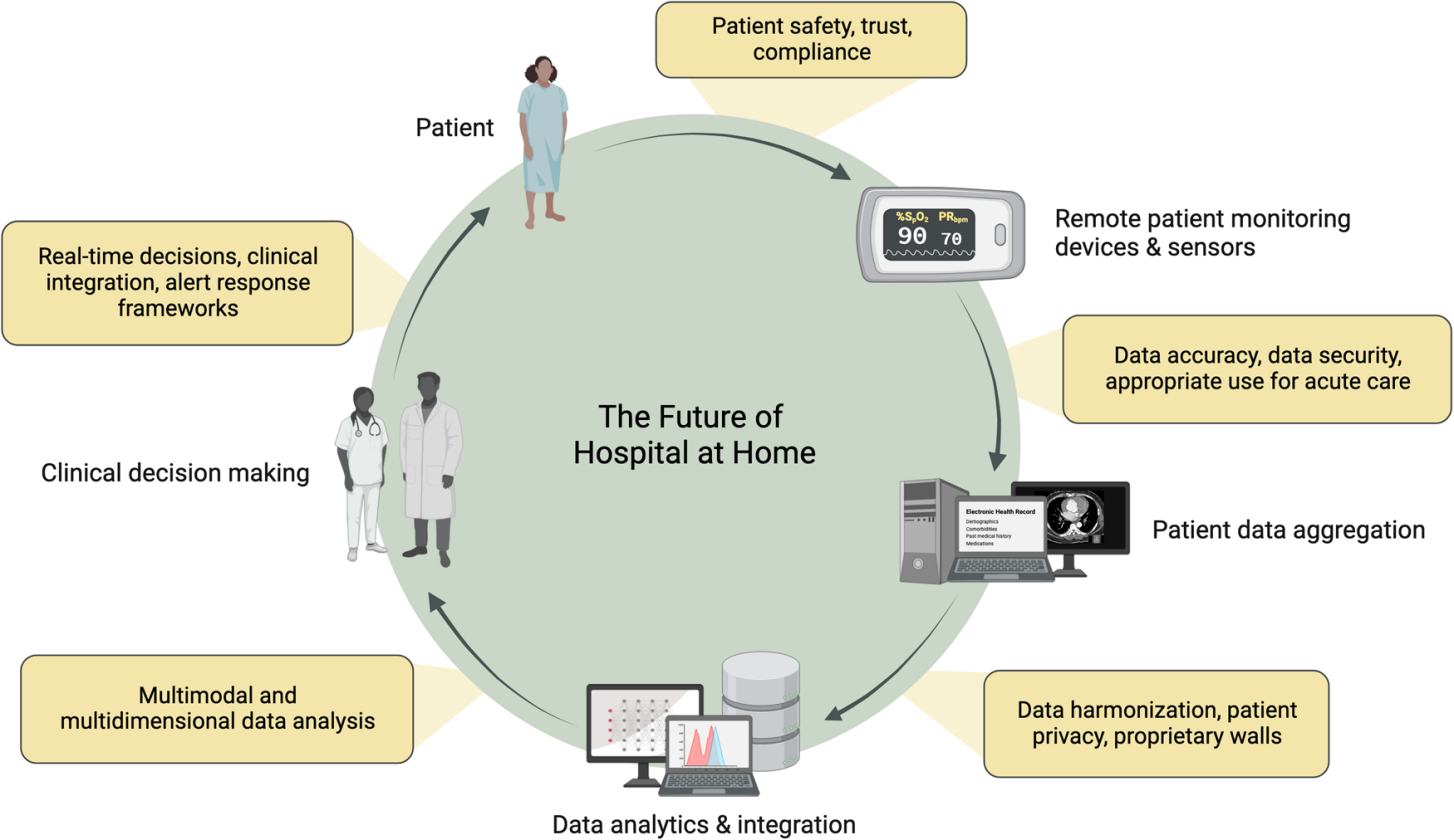
The Future of Hospital at Home. The data life cycle for a future Hospital at Home model with practical and technological barriers for broad implementation. Multimodal patient data is aggregated, analyzed, integrated, interpreted, and applied to provide acute-level care in the home.

Most hospitals in the US digitized their health records after it was mandated by the HITECH Act in 2009^68^. Electronic health records have slowly increased their capabilities, however, while the internal-facing hospital/clinic interface is evolving, the patient and device manufacturer-facing interface leave more to be desired^69^. There are services that help collect home-based digital sensor information and input it into the EHR through an application programming interface (API), usually as a portable document format (PDF) file or a spreadsheet that is typically overlooked during a busy clinic visit^70^. In many countries, tapping the full potential of the EHR is still a large barrier, which is slowly being overcome^71^. There is a glaring need for platforms that ingest this data from an HaH perspective and turn into a personalized clinical decision support tool compliant with local privacy rules to help inform the clinical management plan. Additionally, many current hospital programs have post-discharge follow-up phone call programs. HaH programs should strive to align with current care models as they assess their technical and workforce needs and iterate based on their concerns to effectively extend acute care to the home (Fig. 2).

### Incentive conflict

The US healthcare system is great at managing sick care, but this has had the unfortunate side effect of incentivizing more healthcare visits and procedures. Hospitals are a major lobbying force, and they bring in over $ 1 trillion in revenue in the US per year. The value-based care system has slowly moved the needle towards preventing dollars spent by focusing on dollars saved, but it is not ubiquitous^72^. HaH companies would effectively help reduce hospital utilization and allow hospitals an alternative form of care delivery to address the health problems of our continually aging population and overall population morbidity. While this is ideal, we need more studies to rigorously evaluate and publicize the hard clinical outcomes and cost effectiveness of HaH programs. At a societal level, HaH could help avoid billions in capital costs to build more brick-and-mortar hospitals, without alienating the existing hospital infrastructure. Further, nursing organizations have expressed concern over quality-of-care delivery with HaH programs. Engaging nursing and hospital leaders as stakeholders, understanding their concerns and expectations, and defining their role within the HaH model will be needed to overcome potentially negative views on HaH programs. Overall, there is already an alignment on the desire to keep patients healthy, but there now needs to be more alignment of incentives around keeping the patients that qualify for HaH out of the hospital.

### Privacy and scalability

These are consistently stated problems for any novel health technology and care delivery solution involving transfer of data especially in the context of protected health information (PHI). Fortunately, there have already been significant developments like privacy preserving frameworks, deidentification, developing and updating security and data use infrastructure, and building out governance tokens for large datasets. Scalability was an issue for in-person HaH programs given the reliance on clinical staffing, however, with digital sensors, virtual care delivery platforms and GMAI models to help with data analytics and processing, the concept of acute care with a virtual medical assistant linked to a care delivery team is possible.

While many think that the HaH model is a major paradigm shift in acute care delivery that requires rewiring of the care delivery system, the reality is that the current infrastructure is completely able to deliver this type of care. Hospitals have already started developing digital command centers for both inpatient and outpatient monitoring^73,74^, health systems are already using third party vendors for remote monitoring of outpatient high-risk patients, and the remote monitoring ecosystem has matured to be able to provide real-time clinically actionable insights. None of these challenges are insurmountable and HaH programs already have the tools and evidence to answer these questions that can address quality of care concerns, improve trust, save time and dollars, and ultimately provide more patient-centric care. We believe the ideal program would be one that feels as seamless as an admission to the hospital, with a workforce that is specifically trained to provide culturally compatible care, technology that is vetted to provide clinically actionable insight and programmatic buy-in from the system, community, and individual level.

## Conclusion

HaH models have significant promise with novel innovations in digital sensors, large language models for data processing, and data pipelines built for remote care delivery during COVID-19; it is now time to rigorously evaluate their ability to become a permanent alternative form of acute care management.

## Search strategy and selection criteria

We performed a web-based literature search to identify peer-reviewed publications that substituted inpatient hospital admission with acute-level at-home care. Using the National Center for Biotechnology Information, PubMed, and Medical Subject Heading (Mesh) databases were queried on March 3, 2023. The PubMed search included the following keywords: hospital at home; hospital-at-home; home hospitalization; virtual hospital; acute care at home; and hospital-level care at home. The PubMed queries generated a total of 3 MeSH search terms (hospitalization; hospitals; home environment), and a MeSH database search generated a MeSH Unique ID (D018575) with the Mesh Heading, “home care services, hospital based.” Using this Heading, a PubMed MeSH search was performed, and articles were subsequently filtered by article type. If a PubMed key word search did not generate MeSH terms (acute care at home; hospital-at-home), then a filtered PubMed search was performed. Articles that reported on in-person, virtual and hybrid care models were included. Search filters included the following: US-based, English language; human species; substitutive care for inpatient admission; article types (clinical study, clinical trial, comparative study, controlled clinical trial, evaluation study, multicenter study, observational study, pragmatic clinical trial, randomized controlled trial). Predictive modeling studies, palliative care and mental health applications, meta-analyses, case reports, and systematic reviews were excluded. Furthermore, in preparation for this manuscript, the current authors shared various peer-reviewed HaH-related articles outside of the PubMed and MeSH database queries, and the shared articles that met the above criteria were included.

### Supplementary information

Supplementary Table 1. Summary of peer-reviewed publications on hospital-at-home research studies.

## Data Availability

No datasets were generated or analyzed for the creation of this work.
