# Supplementary material for: The hospital at home in the USA: current status and future prospects
Source: NPJ Digit Med. 2024 Feb 27;7:48. doi: 10.1038/s41746-024-01040-9 (PMC10899639; doi:10.1038/s41746-024-01040-9)
Supplement: Supplementary file 1 — Supplementary Table 1. Summary of peer-reviewed publications on hospital-at-home research studies. [file 41746_2024_1040_MOESM1_ESM.docx]

**Supplementary Table 1. Summary of peer-reviewed publications on US-based hospital-at-home research studies**

| **First Author (Year)** | **Study Type, Delivery of Care** | **n** | **Inclusion Criteria** | **Clinical Indications** | **Intervention** | **Clinical Outcomes** | **Other Outcomes** | **Results** |
| --- | --- | --- | --- | --- | --- | --- | --- | --- |
| Leff, et al. (1999) [1] | PCS, in-person | 17 | - ≥65 years old - Medicare insurance - Required acute hospital admission | - CAP - HF - COPD - Cellulitis | - Initial eval at home by MD - Nursing supervision first 24h then adjusted based on patient needs - Daily visits by MD - MD 24h on call | - Length of stay - Emergency situations - Critical complications | - Health care utilization (e.g., number of physician encounters) - Patient experience - Caregiver experience - Hospital costs/charges | - Similar length of stay - 2 emergency situations in HaH; 1 in inpatient group - 0 critical complications compared to 1 in inpatient group - Lower health care utilization compared to inpatient group - High patient satisfaction - group - Low caregiver burden - Lower hospital charges compared to inpatient group |
| Eron, et al. (2004[)](https://academic.oup.com/cid/article/39/8/1175/296195?login=true) [2] | PCS, virtual | 25 | - Medicare insurance - Required acute hospital admission - Charlson comorbidity index* - Karnofsky index^ | - CAP - Cellulitis - UTI | - Initial video and medical equipment set up at home by member of telemedicine team - MD or NP perform initial televisit to test home equipment - At least one daily televisit with clinician | - Length of stay - Readmission rate - Return to activities of daily living | - Patient experience | - Similar length of stay compared to inpatient group - Fewer readmissions compared to inpatient group - Faster return to activities of daily living compared to inpatient group - Higher patient satisfaction compared to inpatient group |
| Leff, et al. (2005) [3] | PCS, in-person | 455 | - ≥65 years old - Medicare insurance - Required acute hospital admission | - CAP - HF - COPD - Cellulitis | - Initial eval at home or in ED by MD - RN meets patient upon arrival at home - Nursing supervision for initial 8-24h - Daily visits by MD and RN - MD 24h on call | - Length of stay - Emergency situations - Critical and other complications | - Patient experience - Health care utilization (e.g., number of physician encounters) - Hospital costs/charges | - Shorter length of stay compared to inpatient group - Fewer emergency situations compared to inpatient group - Fewer complications compared to inpatient group - High patient and caregiver satisfaction - Lower health care utilization compared to inpatient group - Lower cost compared to inpatient group |
| Leff, et al. (2006) [4] | Same as Leff, et al. (2005) (DOI: 10.7326/0003-4819-143-11-200512060-00008) | | | | | - N/A | - Patient experience | - More likely to be satisfied with treating physician, comfort and convenience of care, admission process, and overall experience compared to inpatient group |
| Leff, et al. (2008) [5] |  |  |  |  |  | - N/A | - Caregiver stress scores | - Less caregiver stress compared to inpatient group |
| Leff, et al. (2009) [6] |  |  |  |  |  | - Activity of daily living measures | - N/A | - Modestly better activities of daily living compared to inpatient group |
| Cryer, et al. (2012) [7] | RR, hybrid | 2728 | - ≥18 years old - Required acute hospital admission | - CAP - CHF - COPD - DVT - Pulmonary embolism - UTI or urosepsis - Cellulitis - Nausea/vomiting - Dehydration | - MD or RN meets patient upon arrival at home - Initial supervision as long as medically necessary, typically 2-3h - Daily visits by MD and RN, with additional RN visits available as needed - Televists by RN available as needed | - Length of stay - Readmission rate - Fall rate - Mortality rate | - Quality-of-care metrics - Health care utilization (e.g., number of physician encounters) - Patient experience - Hospital costs/charges | - Shorter length of stay compared to inpatient group - Similar readmission rate compared to inpatient group - Lower rate of falls compared to inpatient group - Lower mortality rate compared to inpatient group - Similar (high) quality-of-care metrics - Higher patient satisfaction compared to inpatient group - Lower cost compared to inpatient group |
| Summerfelt, et al. (2015) [8] | PCS, hybrid | 102 | - ≥18 years old - Required acute hospital admission | - COPD - CHF - DVT - Asthma - CAP | - LPN meets patient upon arrival at home - Home visits on days 1 and 3 - Daily televisits, alternating between MD and LPN - NP or PA home visits if needed | - Length of stay - Readmission rate - Mortality rate - Activity of daily living measures - Functional status | - Quality-of-care metrics - Health care utilization (e.g., number of physician encounters) - Patient experience | - Similar length of stay compared to inpatient group - Lower readmission rate compared to inpatient group - Higher mortality rate compared to inpatient group - Similar functional outcomes - Similar quality-of-care metrics - Lower health care utilization compared to inpatient group - Higher patient satisfaction compared to inpatient group |
| Federman, et al. (2018) [9] | PCS, hybrid | 507 | - ≥18 years old - Medicare insurance - Required acute hospital admission | - All indications other than those requiring cardiac monitoring or intensive care | - MD or NP meets patient upon arrival at home - Visits by RN at least daily - Visits by MD or NP at least daily with televisit option | - Length of stay - Readmission rate - Transfer to SNF - Referral to home health agency | - Patient experience | - Shorter length of stay compared to inpatient group - Lower readmission rate compared to inpatient group - Lower rate of transfer to SNF compared to inpatient group - Similar rate of referral to home health agency - Higher patient satisfaction compared to inpatient group |
| Levine, et al. (2018) [10] | RCT, hybrid | 21 | - ≥18 years old - Required acute hospital admission | - Any infection - HF - COPD - Asthma | - Daily visits by MD - Twice daily visits by RN - MD 24h on call | - Length of stay - Readmission rate - Physical activity | - Health care utilization (e.g., number of physician encounters) - Patient experience - Hospital costs/charges | - Similar length of stay - Lower readmission rate compared to inpatient group - More physically active compared to inpatient group - Lower health care utilization compared to inpatient group - Similar patient satisfaction - Lower cost compared to inpatient group |
| Mann, et al. (2018) [11] | RR, in-person | 50 | - ≥18 years old - Medicare insurance - Required acute hospital admission | - CAP - COPD - Asthma - HF - Dehydration - UTI | - Daily visits by MD or NP - Daily visits by RN for IV medications - MD or NP 24h on call | - Adverse drug events | - N/A | - 22% of patients experienced adverse drug event |
| DeCherrie, et al. (2019) [12] | RR, in-person | 685 | - ≥18 years old - Medicare/Medicaid insurance - Required acute hospital admission | - Pneumonia - CHF - COPD - Cellulitis - UTI - Asthma - Dehydration - Other | - Daily visits by MD or NP - Visits by RN at least daily - Clinician 24h on call | - Length of stay - Readmission rate - Mortality rate | - Health care utilization (e.g., number of physician encounters) | - Similar length of stay - Lower readmission rate compared to inpatient group - Lower mortality rate compared to inpatient group - Lower health care utilization compared to inpatient group |
| Levine, et al. (2020) [13] | RCT, hybrid | 91 | - ≥18 years old - Required acute hospital admission | - Any infection - HF - COPD - Asthma - CKD - Diabetes - Gout - Hypertensive urgency - AF - Anticoagulation - End of life care | - Daily visits by MD - At least twice daily visits by RN - Televisits with clinical team and specialists as needed | - Length of stay - Readmission rate - Physical activity | - Health care utilization (e.g., number of physician encounters) - Patient experience - Hospital costs/charges | - Lower readmission rate compared to inpatient group - Shorter length of stay compared to inpatient group - More physically active compared to inpatient group - Lower health care utilization compared to inpatient group - Similar patient satisfaction - Lower cost compared to inpatient group |
| Sitammagari, et al. (2021) [14] | PCS, virtual | 1477 | - Positive for COVID-19 - DSCRB-65 score > 2 - No major and ≤3 minor criteria for severe pneumonia* | - COVID-19 - Pneumonia | - Clinician televisit within 12 hours of arrival at home - RN or paramedic home visit within 24h of arrival at home - RN 24h on call - Twice daily RN televisits - Daily paramedic televisits | - Length of stay - ICU admission - Mechanical ventilation - Mortality rate | - N/A | - 12-day median length of stay - 10/184 patients required ICU admission - 1/184 required mechanical ventilation - 0/184 deaths |
| Chou, et al. (2021) [15] | RR, virtual | 391 | - Positive for COVID-19 - DSCRB-65 score > 2 - No major and ≤3 minor criteria for severe pneumonia* | - COVID-19 - Pneumonia | - Clinician televisit within 12 hours of arrival at home - RN or paramedic home visit within 24h of arrival at home - RN 24h on call - Twice daily RN televisits - Daily paramedic televisits | - Care escalation requiring inpatient admission | - N/A | - Patients with low oxygen saturation and higher comorbidity burden had higher risk of care escalation. - Patients with respiratory aberrations and tachycardia at admission had higher rate of immediate care escalation |
| [S](https://www.ncbi.nlm.nih.gov/pmc/articles/PMC6489357/#CR12)iu, et al. (2022) [16] | RR, hybrid | 443 | - ≥18 years old - Medicare insurance - Required acute hospital admission | - All indications other than those requiring cardiac monitoring or intensive care | - MD or NP meets patient upon arrival at home - Visits by RN at least daily - Visits by MD or NP at least daily with televisit option | - Length of stay - Readmission rate - ED revisit rate | - N/A | - Both low and high SES patients had shorter length of stay, lower rate of readmission and lower rate of ED revisit compared to inpatient counterparts |
| Levine, et al. (2022) [17] | RCT, hybrid | 172 | - ≥18 years old - Required acute hospital admission | - Any infection - HF - COPD - Asthma | - Initial eval at home by MD - Daily televisits by MD facilitated by home RN or paramedic - At least twice daily visits by RN - Televisits with clinical team and specialists as needed | - Number of adverse events - Readmission rate | - Patient experience | - Similar number of adverse events, readmission rate and patient satisfaction between in-person and virtual HaH patient groups |
| Hernandez, et al. (2023) [18] | RR, hybrid | 1031 | - ≥18 years old - Required acute hospital admission | - Any infection - HF - COPD - Asthma | - Daily visits by MD - At least twice daily visits by RN - Televisits with clinical team and specialists as needed | - Length of stay - Readmission rate - Mortality rate | - Health care utilization (e.g., number of physician encounters) | - Respiratory patients had shorter length of stay and lower health care utilization compared to other illnesses - Similar readmission and mortality rates between respiratory patients and other illnesses |
| Liu, et al. (2023) [19] | RR, virtual | 2031 | - Positive for COVID-19 - DSCRB-65 score > 2 - No major and ≤3 minor criteria for severe pneumonia* | - COVID-19 - Pneumonia | - Clinician televisit within 12 hours of arrival at home - RN or paramedic home visit within 24h of arrival at home - RN 24h on call - Twice daily RN televisits - Daily paramedic televisits | - Care escalation requiring inpatient admission | - N/A | - Compared to non-Hispanic White patients, non-Hispanic Black patients had a lower risk of care escalation and Hispanics had a higher risk of care escalation - Patients who were older, had a higher number of ED visits within a year of HaH care, and lived in low SES areas had a higher risk of care escalation |
| Paulson, et al. (2023) [20] | RR, hybrid | 686 | - ≥18 years old - Required acute hospital admission | - Decompensated heart failure - COPD - UTI - Other | - Initial eval and set up by paramedic - Home visits on day 1 by MD, NP, or PA - At least twice daily visits by RN or paramedic and supervised virtually by RN | - Length of stay - Readmission rate - Mortality rate | - N/A | - 3-day median length of stay - 9.7% readmission rate (30-day) - 0.6% mortality rate (30-day) |

Supplementary Table 1. Summary of peer-reviewed publications on hospital-at-home research studies. All studies were US-based; included patients who received in-person, virtual or hybrid care; and substituted acute inpatient hospital admission with at-home care. Hybrid care includes both in-person and virtual care. PCS is prospective case series. RR is retrospective review. RCT is randomized controlled trial. PMID is PubMed Identifier. ED is emergency department. SNF is skilled nursing facility. MD is medical doctor. NP is nurse practitioner. RN is registered nurse. LPN is licensed practical nurse. IV is intravenous. COPD is chronic obstructive pulmonary disease. HF is heart failure. DVT is deep vein thrombosis. UTI is urinary tract infection. CKD is chronic kidney disease requiring diuresis. AF is atrial fibrillation with rapid ventricular response. SES is socioeconomic status. Other includes clinical conditions believed to be safely treated at home by the admitting physician. DSCRB-65 is scored based on comorbid disease, oxygen saturation, confusion, respiratory rate ≥30 breaths/min, systolic blood pressure <90 mm Hg or diastolic blood pressure ≤60 mm Hg, age ≥65 years [21].

*Based on the American Thoracic Society/Infectious Diseases Society of America Major and Minor Criteria for Severe Pneumonia (source: <https://www.atsjournals.org/doi/10.1164/rccm.201908-1581ST>)
^Based on the Karnofsky Performance Scale Index (source: <https://ascopubs.org/doi/10.1200/JCO.1984.2.3.187?url_ver=Z39.88-2003&rfr_id=ori:rid:crossref.org&rfr_dat=cr_pub%20%200pubmed>)

References:

1.	Leff B, Burton L, Guido S, Greenough WB, Steinwachs D, Burton JR. Home hospital program: a pilot study. J Am Geriatr Soc. 1999;47(6):697-702.

2.	Eron L, King P, Marineau M, Yonehara C. Treating acute infections by telemedicine in the home. Clin Infect Dis. 2004;39(8):1175-81.

3.	Leff B, Burton L, Mader SL, Naughton B, Burl J, Inouye SK, et al. Hospital at home: feasibility and outcomes of a program to provide hospital-level care at home for acutely ill older patients. Ann Intern Med. 2005;143(11):798-808.

4.	Leff B, Burton L, Mader S, Naughton B, Burl J, Clark R, et al. Satisfaction with hospital at home care. J Am Geriatr Soc. 2006;54(9):1355-63.

5.	Leff B, Burton L, Mader SL, Naughton B, Burl J, Koehn D, et al. Comparison of stress experienced by family members of patients treated in hospital at home with that of those receiving traditional acute hospital care. J Am Geriatr Soc. 2008;56(1):117-23.

6.	Leff B, Burton L, Mader SL, Naughton B, Burl J, Greenough WB, 3rd, et al. Comparison of functional outcomes associated with hospital at home care and traditional acute hospital care. J Am Geriatr Soc. 2009;57(2):273-8.

7.	Cryer L, Shannon SB, Van Amsterdam M, Leff B. Costs for 'hospital at home' patients were 19 percent lower, with equal or better outcomes compared to similar inpatients. Health Aff (Millwood). 2012;31(6):1237-43.

8.	Summerfelt WT, Sulo S, Robinson A, Chess D, Catanzano K. Scalable hospital at home with virtual physician visits: pilot study. Am J Manag Care. 2015;21(10):675-84.

9.	Federman AD, Soones T, DeCherrie LV, Leff B, Siu AL. Association of a Bundled Hospital-at-Home and 30-Day Postacute Transitional Care Program With Clinical Outcomes and Patient Experiences. JAMA Intern Med. 2018;178(8):1033-40.

10.	Levine DM, Ouchi K, Blanchfield B, Diamond K, Licurse A, Pu CT, et al. Hospital-Level Care at Home for Acutely Ill Adults: a Pilot Randomized Controlled Trial. J Gen Intern Med. 2018;33(5):729-36.

11.	Mann E, Zepeda O, Soones T, Federman A, Leff B, Siu A, et al. Adverse drug events and medication problems in "Hospital at Home" patients. Home Health Care Serv Q. 2018;37(3):177-86.

12.	DeCherrie LV, Wajnberg A, Soones T, Escobar C, Catalan E, Lubetsky S, et al. Hospital at Home-Plus: A Platform of Facility-Based Care. J Am Geriatr Soc. 2019;67(3):596-602.

13.	Levine DM, Ouchi K, Blanchfield B, Saenz A, Burke K, Paz M, et al. Hospital-Level Care at Home for Acutely Ill Adults: A Randomized Controlled Trial. Ann Intern Med. 2020;172(2):77-85.

14.	Sitammagari K, Murphy S, Kowalkowski M, Chou SH, Sullivan M, Taylor S, et al. Insights From Rapid Deployment of a "Virtual Hospital" as Standard Care During the COVID-19 Pandemic. Ann Intern Med. 2021;174(2):192-9.

15.	Chou SH, McWilliams A, Murphy S, Sitammagari K, Liu TL, Hole C, et al. Factors Associated With Risk for Care Escalation Among Patients With COVID-19 Receiving Home-Based Hospital Care. Ann Intern Med. 2021;174(8):1188-91.

16.	Siu AL, Zhao D, Bollens-Lund E, Lubetsky S, Schiller G, Saenger P, et al. Health equity in Hospital at Home: Outcomes for economically disadvantaged and non-disadvantaged patients. J Am Geriatr Soc. 2022;70(7):2153-6.

17.	Levine DM, Paz M, Burke K, Beaumont R, Boxer RB, Morris CA, et al. Remote vs In-home Physician Visits for Hospital-Level Care at Home: A Randomized Clinical Trial. JAMA Netw Open. 2022;5(8):e2229067.

18.	Hernandez C, Tukpah AC, Mitchell HM, Rosario NA, Boxer RB, Morris CA, et al. Hospital-Level Care at Home for Patients With Acute Respiratory Disease: A Descriptive Analysis. Chest. 2023;163(4):891-901.

19.	Liu TL, Chou SH, Murphy S, Kowalkowski M, Taylor YJ, Hole C, et al. Evaluating Racial/Ethnic Differences in Care Escalation Among COVID-19 Patients in a Home-Based Hospital. J Racial Ethn Health Disparities. 2023;10(2):817-25.

20.	Paulson MR, Shulman EP, Dunn AN, Fazio JR, Habermann EB, Matcha GV, et al. Implementation of a virtual and in-person hybrid hospital-at-home model in two geographically separate regions utilizing a single command center: a descriptive cohort study. BMC Health Serv Res. 2023;23(1):139.

21.	Dwyer R, Hedlund J, Henriques-Normark B, Kalin M. Improvement of CRB-65 as a prognostic tool in adult patients with community-acquired pneumonia. BMJ Open Respir Res. 2014;1(1):e000038.
